# Predictive value of immune cell counts and neutrophil-to-lymphocyte ratio for 28-day mortality in patients with sepsis caused by intra-abdominal infection

**DOI:** 10.1093/burnst/tkaa040

**Published:** 2021-03-22

**Authors:** Shuangqing Liu, Yuxuan Li, Fei She, Xiaodong Zhao, Yongming Yao

**Affiliations:** 1 Medical school of Chinese PLA, No. 28 Fuxing Road, Haidian District, Beijing 100853, China; 2 Department of Emergency, the Fourth Medical Center of the Chinese PLA General Hospital, No. 51 Fucheng Road, Haidian District, Beijing 100048, China; 3 Trauma Research Center, the Fourth Medical Center of the Chinese PLA General Hospital, No. 51 Fucheng Road, Haidian District, Beijing 100048, China

**Keywords:** Lymphocyte counts, Monocyte counts, Predictive value, Mortality, Sepsis, Intra-abdominal infection

## Abstract

**Background:**

The current study aimed to evaluate the value of immune cell counts and neutrophil-to-lymphocyte ratio (NLR) when attempting to predict 28-day mortality.

**Methods:**

We conducted an observational retrospective study that included consecutive septic patients. Severity scores on the first day and peripheral circulating immune cell counts (at day 1, day 3, day 5 and day 7 of admission) were collected during each patient’s emergency intensive care unit stay. We assessed the associations of peripheral circulating immune cell counts and NLR with the severity of illness. The relationships between 28-day mortality and peripheral circulating immune cell counts and NLR with were evaluated using Cox proportional cause-specific hazards models.

**Results:**

A total of 216 patients diagnosed with sepsis caused by IAI were enrolled. The lymphocyte counts (days 1, 3, 5 and 7) and monocyte counts (days 3, 5 and 7) were significantly lower in non-survivors (n = 72) than survivors (n = 144). The NLR values at each time point were significantly higher in non-survivors. The day 1 lymphocyte counts, as well as the monocyte counts, were significantly lower in the highest-scoring group, when stratified by the Acute Physiology and Chronic Health Evaluation II and Sequential Organ Failure Assessment scores, than in the other groups (*p* < 0.05). The day 1 NLR was significantly higher in the highest-scoring group than in the other groups (*p* < 0.05). The day 5 and day 7 lymphocyte counts, day 3 and day 7 monocyte counts and day 7 NLR were significant predictors of 28-day mortality in the Cox proportional hazards models (day 5 lymphocyte count: hazard ratio, 0.123 (95% CI, 0.055–0.279), *p* < 0.001; day 7 lymphocyte count: hazard ratio, 0.115 (95% CI, 0.052–0.254), *p* < 0.001; day 3 monocyte count: hazard ratio, 0.067 (95% CI, 0.005–0.861), *p* = 0.038; day 7 monocyte count: hazard ratio, 0.015 (95% CI, 0.001–0.158), *p* < 0.001; day 7 NLR: hazard ratio, 0.773 (95% CI, 0.659–0.905), *p* = 0.001).

**Conclusions:**

The results showed that circulating lymphocytes and monocytes were dramatically decreased within 7 days in non-survivors following sepsis from an IAI. Lymphocyte counts, monocyte counts and NLR appeared to be associated with the severity of illness, and they may serve as independent predictors of 28-day mortality in septic patients with IAIs.

Highlights

- An observational retrospective study for seeking prognostic markers in septic patients with intro-abdominal infections (IAI) was carried out.
- The counts of lymphocytes and monocytes are decreased markedly within 7 days in non-survivors with sepsis caused by IAIs.
- The counts of lymphocytes and monocytes in non-survivors were significantly lower than those in survivors with sepsis caused by IAIs.
- The counts of lymphocytes and monocytes, as well as the neutrophil-to-lymphocyte ratio, were independent predictors of 28-day mortality in patients with sepsis caused by IAIs.

## Background

Sepsis is a clinical syndrome caused by an exaggerated immune response to infection that threatens the lives of patients and their quality of life [1–3]. Although hospital mortality related to sepsis has declined, sepsis still affects approximately 31.5 million patients annually, resulting in 5.3 million deaths worldwide each year [4]. Sepsis, as an important global health concern, is still the focus of medical attention and research.

Intra-abdominal infection (IAI) is the second most common cause of infection in the intensive care unit and is often related to prolonged morbidity and a significant mortality rate [5–9]. Approximately 10–15% of IAIs can lead to sepsis and septic shock [10]. IAI occurs due to the invasion and replication of causative organisms in the abdominal cavity [6]. A variety of predisposing factors contribute to the development of IAI, including appendicitis, laparotomy, bowel perforation, intestinal hernias and the insertion of medical devices [6, 11].

Both innate and adaptive immune responses are involved in the development of sepsis. Innate immunity is activated by pathogens as the first line of defense and plays a pivotal role in the initiation of adaptive immunity [12]. The battle between host immunity and pathogens dominates the course of the disease. Numerous studies have reported that refractory opportunistic infections were common in patients who died of sepsis because of immunosuppression [13–15]. Evidence indicates sepsis can affect the function of immune cells, including neutrophils, lymphocytes and monocytes [12, 16]. Currently, the loss and dysfunction of immune cells is considered a major contributing factor to secondary infection and poor prognosis in septic patients [12, 17–19].

Accordingly, alterations in the number and functions of immune cells are potentially associated with mortality in septic patients. The neutrophil-to-lymphocyte ratio (NLR) is a convenient parameter that can be analysed based on a complete blood count [20]. NLR has been reported as an important and effective prognostic marker in multiple diseases, including cancer [21–23], infective endocarditis [24], aneurysmal subarachnoid hemorrhage [25] and inflammatory diseases [26, 27]. Although many studies have investigated the association between NLR and mortality in septic patients, the relationship between NLR and clinical prognosis remains controversial [20, 28, 29]. A large number of studies have focused on the functional changes of immune cells and the alterations of specific immune cell subsets in septic patients [16, 19], but measures of the functions of peripheral circulating immune cells are not routinely available in clinical care, and few studies have investigated the role of peripheral circulating immune cell counts, including neutrophils, monocytes and lymphocytes, in predicting mortality in patients with sepsis. Thus, the present study aimed to explore the dynamic correlations between peripheral circulating immune cell counts, NLR and 28-day mortality in patients with sepsis caused by IAIs.

## Methods

### Participant enrollment and study design

**Figure 1.**
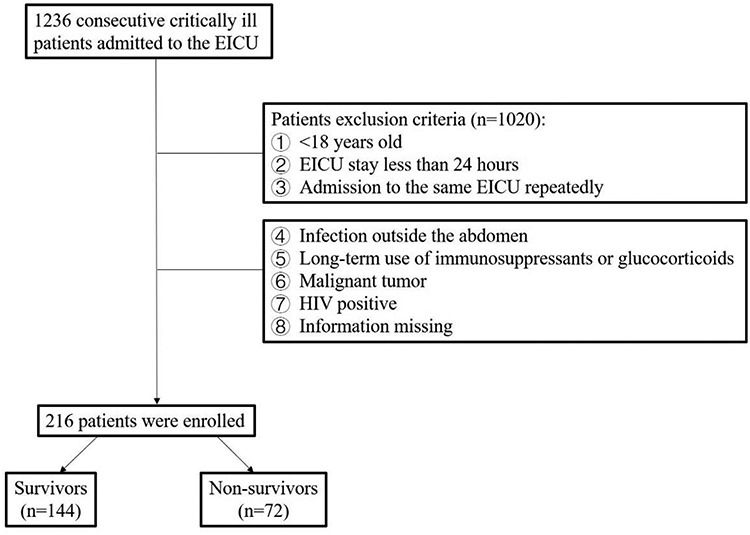
Flowchart of the enrolled patients. *EICU* Emergency Intensive Care Unit

We conducted an observational retrospective study in the Emergency Intensive Care Unit (EICU) of the Fourth Medical Center of the Chinese PLA General Hospital, Beijing, China. From 1 September 2016, to 31 August 2019, 1236 critically ill patients were admitted to the EICU (Figure 1). Adult patients (aged 18 years and above) presenting to the EICU who met the criteria for sepsis 3.0 (the Third International Consensus Definitions for Sepsis and Septic Shock) [30] were enrolled. The exclusion criteria of our study were as follows: (1) <18 years old; (2) EICU stay <24 hours; (3) repeated admission to the same EICU; (4) extra-abdominal infections; (5) long-term use of immunosuppressants or glucocorticoids; (6) malignant tumors; (7) HIV-positive status; and (8) no available information. All patients in our study were observed for at least 28 days.

All patients received 24-hour medical care and the management followed the instructions of the Surviving Sepsis Campaign Guideline, with the goal of initial resuscitation and infection control [30]. The study complied with the Declaration of Helsinki, and the study protocol was approved by the Human Ethical Committee of the Fourth Medical Center of the Chinese PLA General Hospital, Beijing, China.

### Definitions

IAI is defined as an infection in the abdominal cavity, presenting with the clinical signs of local and systemic inflammation (pain, tenderness, fever, tachycardia and tachypnea) [31, 32]. IAIs can be classified according to the time and severity of the infection [32]. Nosocomial IAIs are defined as infections occurring 48 hours after admission for reasons other than IAI; other IAIs are classified as being of community-onset. Simple or uncomplicated IAIs occur when a single organ is involved, whereas complicated IAIs extend beyond the source organ and involve multiple intra-abdominal viscera.

### Data collection

We collected the data from the electronic and paper medical records of patients. Data regarding demographics, vital signs, comorbidities, Sequential Organ Failure Assessment (SOFA) scores (day 1 post-admission), Acute Physiology and Chronic Health Evaluation (APACHE) II scores (day 1 post-admission), laboratory results, the source of the IAI and primary outcomes were recorded on a previously designed e-sheet.

### Laboratory parameter assay

The routine blood tests were measured on consecutive days in our EICU. The counts of circulating immune cells were obtained from routine blood tests. Peripheral blood samples were collected in EDTA for routine blood tests using an XN-10 (B4) automated hematology analyser (Sysmex, Kobe, Japan). The normal ranges for these cell counts are as follows: neutrophils, 2.0–7.5 × 10^9^/L; lymphocytes, 1.0–4.5 × 10^9^/L; and monocytes, 0.2–0.8 × 10^9^/L (data from our laboratory).

### Statistical analysis

The Kolmogorov–Smirnov test was used to analyse the theoretical distribution of the continuous variables. Normally distributed continuous data are reported as the mean ± SD, and non-normally distributed data are shown as the median (interquartile range). Categorical data are expressed as the number (percentage). Comparisons of two groups were performed using the Chi-squared test and the *t* test for independent samples, while comparisons of more than two groups were performed using one-way analysis of variance (ANOVA) followed by the least significant difference (LSD) multiple comparison test. The rank sum test was used to compare non-normally distributed data as follows: the Mann–Whitney *U* test was used for the comparison of two groups and the Kruskal–Wallis one-way ANOVA (k samples) was used for the comparison of more than two groups. Changes in the neutrophil count, monocyte count, lymphocyte count and NLR over time between the survivors and non-survivors were compared with multiple ANOVA. For 28-day mortality, the data were compared using hazard ratios within 95% CIs in the univariate analysis. Multivariate Cox proportional hazards regression models were used to confirm the relationship between peripheral circulating immune cell count-related parameters and 28-day mortality in model 1 and model 2 (adjustment for age, sex, SBP (systolic blood pressure), body weight, APACHE II scores and SOFA scores). The predictive accuracy of peripheral circulating immune cell count-related parameters to detect adverse outcomes was evaluated by the receiver operating characteristic (ROC) method. We compared the area under the curve (AUC) values of different groups using the method of DeLong et al. [33]. All statistical analyses were performed using SPSS® (version 22.0, IBM, USA) and MedCalc® (version 11.4.40, MedCalc Software Ltd, Belgium). Statistical significance was set at *p* <0.05.

## Results

### Baseline clinical characteristics

A total of 1236 patients with sepsis were admitted to the EICU during the study period. The baseline characteristics of the 216 patients who met the inclusion criteria are shown in Table 1. Among them, 144 patients (66.7%) survived >28 days. The vital signs between the two groups were similar on the day of admission, except SBP (*p* < 0.05). A total of 60 patients progressed to hemodynamic instability or septic shock, of whom 41 patients died (*p* < 0.01) (Table S1). The lowest mean arterial pressure (MAP) values of patients with septic shock on the day of diagnosis are available in Table S1. Additionally, there was no significant difference between patients who did and did not survive septic shock with respect to MAP.

**Table 1:** Baseline characteristics of the 216 patients with sepsis caused by intro-abdominal infections

	**Total (n = 216)**	**Survivors (n = 144)**	**Non-survivors (n = 72)**	***P* value**
**Demographics**				
Age, years	54.70 ± 12.877	53.83 ± 12.898	56.43 ± 12.750	0.163
Male, n (%)	116 (53.7)	76 (52.8)	40 (55.6)	0.700
**Vital signs**				
Temperature, °C	38.04 ± 0.886	37.95 ± 0.920	38.24 ± 0.784	0.022
SBP, mmHg	109.37 ± 17.042	113.27 ± 18.082	101.58 ± 11.340	<0.001
Heart rate, bpm	104.76 ± 13.706	104.14 ± 14.578	106.01 ± 11.765	0.344
**Source of intra-abdominal infection, n (%)**				
Gastroduodenal perforation	46 (21.3)	26 (18.1)	20 (27.8)	0.100
Liver abscess	14 (6.5)	12 (8.3)	2 (2.8)	0.204
Appendicitis	68 (31.5)	68 (47.2)	0 (0)	<0.001
Severe acute pancreatitis	45 (20.8)	21 (14.6)	24 (33.3)	0.001
Intestinal perforation	43 (19.9)	17 (11.8)	26 (36.1)	<0.001
**Comorbidities, n (%)**				
Hypertension	41 (19.0)	25 (17.4)	16 (22.2)	0.390
IHD	20 (9.3)	14 (9.7)	6 (8.3)	0.740
COPD	7 (3.2)	6 (4.2)	1 (1.4)	0.497
Autoimmune disease	10 (4.6)	7 (4.9)	3 (4.2)	1.000
CKD	2 (0.9)	0 (0)	2 (2.8)	0.209
**Severity of illness**				
APACHE II scores	22.27 ± 6.55	18.92 ± 3.37	28.97 ± 6.22	<0.001
SOFA scores	8 (6–11)	7 (5–9)	11.5 (8–15.75)	<0.001

*SBP* systolic blood pressure, *IHD* ischemic heart disease, *COPD* chronic obstructive pulmonary disease, *CKD* chronic kidney disease, *APACHE* Acute Physiology and Chronic Health Evaluation, *SOFA* Sequential Organ Failure Assessment. Data are expressed as the mean ± SD, median (interquartile range), or number (%). A *p* value <0.05 indicates statistical significance

The distribution of the source of IAI in the two groups is also presented in Table 1. Overall, appendicitis was identified in 68 (31.5%) patients, followed by gastroduodenal perforation (21.3%), severe acute pancreatitis (20.8%), intestinal perforation (19.9%) and liver abscess (6.5%). Among the survivors, appendicitis accounted for the highest proportion (47.2%). In the non-survivors, intestinal perforation (36.1%) and severe acute pancreatitis (33.3%) were diagnosed in more patients (*p* < 0.05). The constituent ratios of comorbidities in the two groups were similar. The non-survivors were more severely ill on EICU admission, as reflected by higher APACHE II and SOFA scores than the survivors (*p* < 0.01).

During the study period, 361 isolates from 216 patients with microbiologically proven IAI were identified (Table S2). There were no significant differences in the distributions of the microorganisms between the survivors and non-survivors in aerobes, anaerobes or fungi (no viral or parasitic infections). However, the infection rates of *Klebsiella spp.*, *Pseudomonas aeruginosa (P. aeruginosa), Candida albicans (C. albicans)* and *Candida spp. other than C. albicans* in the survivors were significantly lower than those in the non-survivors (*p* < 0.05) (Table S2).

### Kinetic changes in circulating immune cell counts and NLR in sepsis

**Figure 2.**
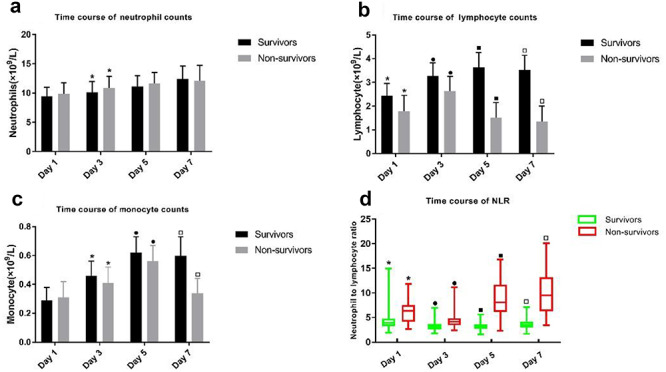
Time course of immune cell counts and neutrophil-to-lymphocyte ratio (NLR). All the data including neutrophil count **(a)**, lymphocyte count **(b)** and monocyte count **(c)** were obtained by routine blood tests, and the NLR **(d)** was obtained indirectly by the ratio of neutrophil count to lymphocyte count. Differences between two groups with the same symbols were considered statistically significant at *p* < 0.05

Neutrophil counts increased over time (from day 1 to day 7) in both survivors and non-survivors (Figure 2a). The overall differences in the time course of neutrophil counts were significant except for the difference between the day 5 and day 7 neutrophil counts in non-survivors (*p* = 0.077). We also compared the neutrophil counts between survivors and non-survivors. There was no significant difference between the two groups as a whole (*p* = 0.053), and only the day 3 neutrophil count in the survivors was significantly lower than that in non-survivors. The lymphocyte counts were elevated in survivors from day 1 to day 5, while they were slightly decreased on day 7 (*p* = 0.131). The lymphocyte counts in non-survivors increased from day 1 to day 3 and then markedly decreased from day 5 of admission (*p* < 0.01). There were significant differences in the lymphocyte counts between survivors and non-survivors at each time point (*p* < 0.01) (Figure 2b). Similarly, the monocyte counts were increased in survivors from day 1 to day 5, but were slightly decreased on day 7 (*p* = 0.054). The monocyte counts in non-survivors were significantly elevated from day 1 to day 5, and then markedly decreased on day 7 (*p* < 0.01). There were significant differences in the monocyte counts between survivors and non-survivors as a whole, and the monocyte counts from day 3 to day 7 of admission in survivors were significantly higher than those in non-survivors (Figure 2c).

As shown in Figure 2d, NLR decreased in survivors from day 1 to day 5 and then increased on day 7 post-admission; there were no statistically significant differences between the NLR values on day 3 and day 5 (*p* = 0.724). The overall differences in the time course of the NLR value were significant in non-survivors. The NLR values in non-survivors were significantly higher than those in survivors at each time point.

### Correlations of immune cell counts and NLR with the severity of illness in septic patients

To evaluate the impact of peripheral circulating immune cell counts on the severity of illness, we compared the cell counts on day 1 of admission among groups categorized by the APACHE II and SOFA scores (Table 2). Based on previous sepsis studies [34, 35], we separated the patients into three groups according to the scoring systems: (1) APACHE II scores: <16, 17–24 and > 24; and (2) SOFA scores: 2–5, 6–10 and > 10. We did not detect any statistically significant differences in neutrophil counts between any two groups. The lymphocyte counts in the highest-scoring group (APACHE II and SOFA scores) were significantly lower, while the NLR values in the highest-scoring group (APACHE II or SOFA scores) were significantly higher than those in the other two groups. Moreover, the monocyte count in the APACHE II < 16 group was significantly higher than that in the APACHE II > 24 group. No significant difference in monocyte counts was noticed in any two groups when stratified according to the SOFA scores. The distributions of survivors and non-survivors according to the APACHE II and SOFA scores are shown in Table S3. The proportions of non-survivors in the highest-scoring groups (59.7% *vs* 16.0% for the APACHE II score; 48.6% *vs* 17.4% for the SOFA score) were significantly larger than those of survivors (*p* < 0.05).

**Table 2:** Comparing the peripheral circulating immune cell counts of studied patients among different groups

**Parameters**	**APACHE II scores**			**SOFA scores**		
	<16	17–24	>24	2–5	6–10	>10
	n = 38	n = 112	n = 66	n = 52	n = 104	n = 60
Neutrophil (×10^9^/L)	9.24 ± 1.73	9.69 ± 1.49	9.70 ± 1.84	9.28 ± 1.49	9.66 ± 1.73	9.83 ± 1.61
Lymphocyte (×10^9^/L)	2.39 ± 0.55^*^	2.37 ± 0.56^†^	1.87 ± 0.71^*, †^	2.32 ± 0.51^‡^	2.31 ± 0.67^§^	1.98 ± 0.69^‡, §^
Monocyte (×10^9^/L)	0.32 ± 0.11	0.29 ± 0.09	0.28 ± 0.08	0.30 ± 0.06	0.30 ± 0.11	0.29 ± 0.09
NLR	3.96 (3.15–4.59)^¶^	4.07 (3.34–4.99)^**^	5.85 (3.95–7.35)^¶, **^	4.09 (3.37–4.73)^††^	4.17 (3.32–5.04)^‡‡^	5.34 (3.75–7.33)^††, ‡‡^

Data are expressed as mean ± SD or median (interquartile range). Differences between two groups with the same symbols were considered statistically significant at *p* values of <0.05

*APACHE* Acute Physiology and Chronic Health Evaluation, *SOFA* Sequential Organ Failure Assessment, *NLR* neutrophil-to-lymphocyte ratio

**Table 3:** Univariate and multivariate Cox regression models to predict 28-day mortality

**Time**	**Parameters**	**Univariate**		**Multivariate (model 1)**		**Multivariate (model 2)** ^a^	
		Hazard ratio (95% CI)	*P* value	Hazard ratio (95% CI)	*P* value	Hazard ratio (95% CI)	*P* value
Day 1	Neutrophil	1.102 (0.961–1.265)	0.166	1.077 (0.841–1.380)	0.558	0.920 (0.724–1.170)	0.498
	Lymphocyte	0.221 (0.147–0.331)	<0.001	0.143 (0.044–0.463)	0.001	0.771 (0.260–2.289)	0.640
	Monocyte	7.068 (0.940–53.111)	0.057	1.764 (0.293–10.627)	0.535	0.551 (0.063–4.846)	0.591
	NLR	1.287 (1.199–1.381)	<0.001	0.879 (0.644–1.200)	0.417	1.197 (0.878–1.632)	0.257
Day 3	Neutrophil	1.163 (1.017–1.331)	0.028	1.182 (0.934–1.496)	0.165	1.111 (0.889–1.389)	0.355
	Lymphocyte	0.256 (0.175–0.376)	<0.001	0.135 (0.050–0.364)	0.000	0.379 (0.143–1.008)	0.052
	Monocyte	0.022 (0.002–0.253)	0.002	0.047 (0.004–0.490)	0.011	0.067 (0.005–0.861)	0.038
	NLR	1.486 (1.318–1.676)	<0.001	0.690 (0.423–1.124)	0.136	0.823 (0.529–1.281)	0.388
Day 5	Neutrophil	1.110 (0.964–1.277)	0.147	1.110 (0.927–1.328)	0.256	1.011 (0.829–1.234)	0.912
	Lymphocyte	0.196 (0.147–0.261)	<0.001	0.088 (0.044–0.177)	0.000	0.123 (0.055–0.279)	<0.001
	Monocyte	0.020 (0.003–0.150)	<0.001	0.620 (0.061–6.281)	0.685	0.205 (0.017–2.411)	0.207
	NLR	1.375 (1.304–1.449)	<0.001	0.797 (0.669–0.949)	0.011	0.833 (0.691–1.005)	0.056
Day 7	Neutrophil	0.949 (0.859–1.047)	0.298	1.115 (0.980–1.269)	0.099	1.072 (0.927–1.240)	0.349
	Lymphocyte	0.218 (0.166–0.286)	<0.001	0.090 (0.046–0.178)	0.000	0.115 (0.052–0.254)	<0.001
	Monocyte	0.000 (0.000–0.001)	<0.001	0.010 (0.001–0.100)	0.000	0.015 (0.001–0.158)	<0.001
	NLR	1.247 (1.199–1.296)	<0.001	0.747 (0.650–0.857)	0.000	0.773 (0.659–0.905)	0.001

A *p* value <0.05 indicates statistical significance

^a^Model 2 included age, sex, systolic blood pressure, body weight, Acute Physiology and Chronic Health Evaluation II scores and Sequential Organ Failure Assessment scores

*NLR* neutrophil-to-lymphocyte ratio

Furthermore, we also compared the peripheral immune cell counts between survivors and non-survivors who received glucocorticoids for adrenal insufficiency (Table S4). The lymphocyte and monocyte counts in non-survivors were significantly lower than those in survivors (*p* < 0.01). There was no significant difference in the neutrophil count between survivors and non-survivors (*p* = 0.701).

### Peripheral circulating immune cell counts, NLR and 28-day mortality

In the univariate Cox proportional hazards model (Table 3), day 3 neutrophil count, day 1 to day 7 lymphocyte count, day 3 to day 7 monocyte count and day 1 to day 7 NLR were associated with 28-day mortality. In the multivariate Cox proportional hazards model 1, day 1 to day 7 lymphocyte count, day 3 and day 7 monocyte count and day 5 and day 7 NLR were associated with 28-day mortality. After adjusting for age, sex, SBP, body weight, APACHE II score and SOFA score, day 5 and day 7 lymphocyte count, day 3 and day 7 monocyte count and day 7 NLR were found to be independent predictors of 28-day mortality in patients with sepsis (Table 3).

**Figure 3.**
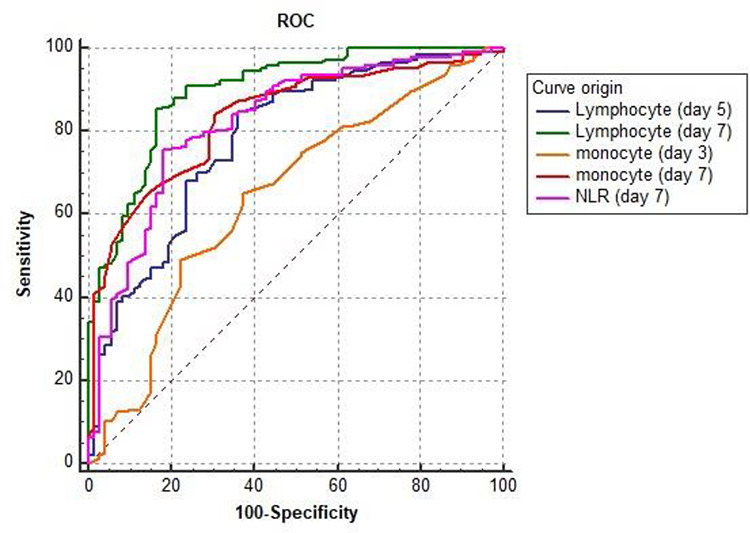
The receiving operating characteristic (ROC) analysis of peripheral circulating immune cell counts and neutrophil-to-lymphocyte ratio (NLR) for predicting the development of 28-day mortality

ROC curves were calculated to investigate the predictive value of peripheral circulating immune cell counts and NLR in septic patients (Figure 3). The ROC curve analyses revealed that the AUC values for predicting 28-day mortality were 0.783 for day 5 lymphocyte count (95% CI, 0.722–0.836), 0.897 for day 7 lymphocyte count (95% CI, 0.848–0.934), 0.644 for day 3 monocyte count (95% CI, 0.577–0.708), 0.834 for day 7 monocyte count (95% CI, 0.778–0.881) and 0.823 for day 7 NLR (95% CI, 0.765–0.871). The best clinical cut-off value, sensitivity and specificity for each parameter are shown in Table 4. The results showed that day 7 lymphocyte and monocyte counts were promising parameters with good sensitivity, specificity and Youden index values in patients with sepsis induced by IAIs.

**Table 4:** Receiver operating characteristic analysis of factors independently predicting 28-day mortality of sepsis caused by intra-abdominal infection

**Parameters**	**AUC**	**95% CI**	**Cut-off point**	**Sensitivity (%)**	**Specificity (%)**	**Youden index**
Lymphocyte (Day 5)	0.783	(0.722–0.836)	3.06	84.72	63.89	0.49
Lymphocyte (Day 7)	0.897	(0.848–0.934)	2.99	85.42	83.33	0.69
Monocyte (Day 3)	0.644	(0.577–0.708)	0.42	65.28	62.5	0.28
Monocyte (Day 7)	0.834	(0.778–0.881)	0.48	84.03	69.44	0.53
NLR (Day 7)	0.823	(0.765–0.871)	4.18	75.69	81.94	0.58

The comparison of receiver operating characteristic AUC was analysed by the method of DeLong et al. [33].

Differences between two groups with the same symbols were considered statistically significant at *p* values of < 0.05

*AUC* area under the curve, *NLR* neutrophil-to-lymphocyte ratio

## Discussion

It has been demonstrated that the immune system plays a pivotal role in the pathogenesis of septic complications [12]. Changes in the numbers of immune cells have a great impact on the direction of the body's inflammatory response in different stages of sepsis. In the present study, the clinical significance of peripheral circulating immune cells in sepsis caused by IAI was assessed. The results indicate that circulating immune cells might be involved in immune dysfunction and further determine the outcomes of septic patients.

It is well known that a wide range of patient factors are associated with sepsis mortality, including age, sex, blood pressure, immune status and comorbidity [36]. It was found that the SBP in non-survivors was significantly lower than those in survivors, implying that septic shock may be the underlying cause of death in non-survivors. As the most severe form of sepsis, septic shock is associated with poor outcomes and a high mortality rate of up to 50%, and is characterized by persistent tissue hypoperfusion [37]. Regarding MAP, a very important monitoring indicator for septic shock, it was reported that a MAP level higher than 60 mmHg may be required and proposed [38]. In our study, 56.9% of the non-survivors suffered from septic shock and died within 28 days, despite their MAP levels being similar to those of the surviving septic shock patients (Table S1). Thus, discussions regarding the level of intervention for septic shock are necessary. Intravenous injection of hydrocortisone at a dose of 200 mg per day is recommended for those with adrenal insufficiency to restore hemodynamic stability [39]. In our study, the counts of lymphocytes and monocytes in septic shock patients receiving glucocorticoids were found to be much lower in non-survivors, indicating that lymphopenia and monocytopenia in non-survivors were profound and hard to reverse despite the use of glucocorticoids. In addition, sepsis is an infectious condition manifesting with the presence of bacteria, fungi, parasites or viruses in the bloodstream [40]. The dreadful outcomes of septic patients are associated with dysregulated immune defenses and complex microbiome–host interactions. *P. aeruginosa* and *C. albicans* are common opportunistic pathogens that play a crucial role in the pathogenesis of sepsis; they often cause systemic infections and are associated with immunosuppression [41]. Higher levels of infection with *P. aeruginosa* and *C. albicans* were found in the non-survivors in the current study (Table S2), which demonstrates that the immune status is an important factor of mortality in sepsis.

There is growing evidence that lymphopenia (T, B and natural killer cells) often occurs in the course of sepsis and further results in immunoparalysis [42]. Previous studies reported that lymphocyte counts decreased at the onset of sepsis and could maintain a stable low level for up to 28 days [43, 44]. Drewry et al. [43] conducted a single-center, retrospective cohort study to determine whether persistent lymphopenia on the fourth day following the diagnosis of sepsis predicted mortality. They found that the absolute lymphocyte counts were significantly lower in non-survivors than in survivors over the 4-day period following the diagnosis of sepsis. Likewise, the main risk factor for sepsis-induced death in elderly patients was prolonged lymphopenia [45]. In the current study, we found that the lymphocyte counts in non-survivors were significantly lower than those in survivors at each observational time point, which was in agreement with the findings of previous reports [43, 45]. Interestingly, our study showed that the lymphocyte counts increased on day 3 then decreased and stayed low instead of showing a persistent downward trend in non-survivors. These findings suggest that the immune system might boost immunity by increasing the number of lymphocytes in the peripheral bloodstream in the early stage of sepsis, which could be beneficial to host survival. However, a dramatic reduction in circulating lymphocytes on day 5 in non-survivors was observed in this study, which could be explained by the exhaustion and depletion (apoptosis) of lymphocytes due to an overwhelming inflammatory response [46]. Thus, lymphocytes appear to be consumed largely within a week, which is partly attributable to immune disorders in the setting of sepsis. In addition, the immune functional changes might be reflected partly by the alteration in lymphocyte counts in the peripheral bloodstream, and an improvement in the host immunity of critically ill patients can be considered if the lymphocyte counts return to the normal range.

We further evaluated the dynamic changes in monocyte counts, neutrophil counts and NLR over the 7-day period in our study. It was observed that the monocyte counts continued to rise from day 1 to day 5, both in survivors and non-survivors, dropping markedly in non-survivors on day 7. The monocyte counts in non-survivors were lower than those in survivors from day 3 to day 7, which was consistent with the findings of other studies [47, 48]. The trend of monocyte counts was similar to that of lymphocyte counts in septic patients. Monocytes are essential for fighting off invading bacteria as the first interceptors via phagocytosis and immune processing. Recently, transient but profound monocytopenia was observed in a human experimental endotoxemia model. The early release of classical monocytes from bone marrow within 25 hours could facilitate the recovery of circulating monocytes [49], which might explain why monocytes increased in the early stage of sepsis in our study. With regard to the neutrophil count, we noted that this was elevated over time in both survivors and non-survivors, but there was no significant difference between the two groups. A possible explanation for this finding is that neutrophils are the most abundant subpopulation of leucocytes as the first line of defense against invading pathogens [1], and the mechanisms affecting the number of neutrophils are complex and various, including stress, infection, antibiotic exposure and the hemopoietic function of bone marrow. Therefore, the number of neutrophils is hardly reduced solely by one single factor (such as infection). NLR can serve as a convenient biomarker in various diseases. Herein, the NLR values in non-survivors were significantly higher than those in survivors, and the difference in NLR values between the two groups was largely attributable to the lymphocyte response.

To further assess the relationship between peripheral circulating immune cells and the severity of illness, the patients were divided into several groups according to their APACHE II scores or SOFA scores. It was observed that the numbers of lymphocytes and monocytes were significantly lower in the highest-scoring group, indicating that the numbers of lymphocytes and monocytes were correlated with the severity of illness. The NLR value in the highest-scoring group was higher than that in the other groups, while the neutrophil count did not differ significantly according to the scoring system. Similar findings have previously been reported, showing the possible association between high NLR and sepsis-related mortality [26, 27, 43].

The current study was performed to investigate the factors that independently predict 28-day mortality in patients with sepsis caused by IAI. Our data showed that day 5 and day 7 lymphocyte counts, day 3 and day 7 monocyte counts and day 7 NLR were independent predictors of 28-day mortality in septic patients after adjustment for age, sex, SBP, body weight, APACHE II score and SOFA score, using Cox multivariate models. These results are consistent with those of other studies [43, 50]. Moreover, we used ROC curves to analyse the predictive efficacy of each of the above independent factors for 28-day mortality in sepsis. The results revealed that the day 7 lymphocyte count had a relatively large AUC and relatively high sensitivity. From a previous report, a lower day 3 lymphocyte count was found to be correlated with higher mortality and served as an independent risk factor, which was similar to the finding in our study [51]. Overall, the reduction in lymphocytes has important predictive value for the mortality of patients with sepsis.

From the present study, the day 7 NLR was closely associated with 28-day mortality, with an AUC of 0.823 (sensitivity, 75.69%; specificity, 81.94%). The cut-off point of NLR was 4.18, which was consistent with the optimal cut-off point of a previous study (4.36) [52]. Similarly, the day 3 and day 7 monocyte counts were also shown to have predictive value for 28-day mortality in sepsis secondary to IAI. Chung et al. [47] compared the changes in monocyte counts between survivors and non-survivors and found that non-survivors showed a significant decrease in monocyte counts from the premorbid state to sepsis; conversely, a significant increase was noted in survivors. Collectively, monocyte counts appear to be independently associated with the fatal outcome of septic complications.

This research has several strengths. First, the sample size of our primary cohort was relatively large, which reduces selection bias. Second, we dynamically monitored the parameters to summarize the complete process of data variation, unlike most studies that only checked one single time point. Third, to the best of our knowledge, this study is the first to find that lymphocytes and monocytes increase initially and then decrease significantly within a week from the onset of sepsis caused by IAI. Our results imply that early monitoring and intervention are crucial for septic patients. Finally, unlike previous reports [43, 50], we note that peripheral circulating immune cells rarely fall below the lower limit of the normal range, even in patients with critical illnesses. Therefore, the decreased quantity and trend of circulating immune cells might have more predictive value for 28-day mortality in patients with sepsis caused by IAI than the cell count itself.

Nevertheless, this study has several limitations. First, our study is a retrospective, observational study conducted at a single study center. A prospective multicenter study is needed to further validate our results. From this investigation, we could not conclude that the decrease in peripheral circulating immune cells reflects dysfunction of the immune system. Moreover, the precise mechanism underlying the reduction in peripheral circulating immune cells could not be elucidated in our study. Second, we could not investigate other time points to cover the entire course of sepsis, and the serial changes in peripheral circulating immune cells for more than a week were still ambiguous. Thus, it is important for us to further understand the characteristics of sepsis in more detail. Third, some patients often received several routine blood tests on the same day, and we only used the first test to ensure data consistency. However, we could inevitably miss important information on cell count alterations. Finally, we only determined the relationship between immune cell counts and mortality in septic patients. Inflammatory biomarkers (such as procalcitonin or interleukin-6) were not assessed in our study. It is well known that an important feature of sepsis is the complex cytokine network, including pro-inflammatory and anti-inflammatory cytokines, which thereby leads to overwhelming tissue and organ injury due to the dysregulation of the immune response to acute insults [1, 2, 28]. Thus, this is the main subject of our forthcoming study.

## Conclusions

In conclusion, the present study confirmed that lymphocytes, monocytes and NLR are associated with the severity of illness in septic patients. Our data provide interesting insight into the pathophysiology of sepsis, and peripheral circulating immune cell counts and NLR appear to be independent predictors of 28-day mortality in patients with sepsis caused by IAI.

## Supplementary Material

Supplementary_materials-Shuangqing_Liu_tkaa040

Editing_Certificate_tkaa040
