# Supplementary material for: Predictive value of immune cell counts and neutrophil-to-lymphocyte ratio for 28-day mortality in patients with sepsis caused by intra-abdominal infection
Source: Burns Trauma. 2021 Mar 22;9:tkaa040. doi: 10.1093/burnst/tkaa040 (PMC7982795; doi:10.1093/burnst/tkaa040)
Supplement: Supplementary_materials-Shuangqing_Liu_tkaa040 [file supplementary_materials-shuangqing_liu_tkaa040.docx]

Table S1. Distributions of septic shock patients and mean arterial pressures between the two groups.

| Parameters | Total | Survivors | Non-survivors | *P* value |
| --- | --- | --- | --- | --- |
|  | (n=216) | (n=144) | (n=72) |  |
| Septic shock (n, %) | 60 (27.8) | 19 (13.2) | 41 (56.9) | <0.01 |
| Mean arterial pressure (mmHg) | 54.82±7.84 | 56.26±8.04 | 54.17±7.76 | 0.338 |

Note: mean arterial pressure (MAP) indicated the MAP in septic shock patients in the two groups. *P*-value <0.05 indicated statistical significance.

Table S2. Distributions of the microorganisms isolated from septic patients with IAIs.

| Microorganisms | Total | Survivors | Non-survivors | *P* value |
| --- | --- | --- | --- | --- |
|  | (n=361) | (n=211) | (n=150) |  |
| **Aerobes** |  |  |  |  |
| Gram-negative bacteria | 289 (80.1) | 167 (79.1) | 122 (81.3) | 0.608 |
| Escherichia coli | 165 (45.7) | 92 (43.6) | 73 (48.7) | 0.341 |
| Klebsiella spp. | 47 (13.0) | 38 (18.0) | 9 (6) | 0.001 |
| Pseudomonas aeruginosa | 32 (8.9) | 10 (4.7) | 22 (14.7) | 0.001 |
| Citrobacter spp. | 16 (4.4) | 9 (4.3) | 7 (4.7) | 0.855 |
| Proteus mirabilis | 13 (3.6) | 10 (4.7) | 3 (2) | 0.169 |
| Morganella morganii | 10 (2.8) | 6 (2.8) | 4 (2.7) | 1.000 |
| Serratia spp. | 5 (1.4) | 2 (0.9) | 3 (2) | 0.699 |
| Enterobacter spp. | 1 (0.3) | 0 (0) | 1 (0.7) | 0.416 |
| Gram-positive bacteria | 22 (6.1) | 8 (3.8) | 14 (9.3) | 0.030 |
| Enterococcus spp. | 9 (2.5) | 2 (0.09) | 7 (4.7) | 0.059 |
| Streptococcus spp. other than S. pneumoniae | 4 (1.1) | 3 (1.4) | 1 (0.07) | 0.869 |
| Staphylococcus aureus | 4 (1.1) | 1 (0.5) | 3 (2) | 0.393 |
| Coagulase-negative Staphylococcus | 3 (0.8) | 0 (0) | 3 (2) | 0.140 |
| Gram-positive cocci, other | 2 (0.6) | 2 (0.9) | 0 (0) | 0.513 |
| **Anaerobes** |  |  |  |  |
| Anaerobes, total | 17 (4.7) | 10 (4.7) | 7 (4.7) | 0.974 |
| Bacteroides spp. | 10 (2.8) | 6 (2.8) | 4 (2.7) | 1.000 |
| Clostridium spp. | 5 (1.4) | 2 (0.9) | 3 (2) | 0.699 |
| Anaerobes, other | 2 (0.6) | 2 (0.9) | 0 (0) | 0.513 |
| **Fungi** |  |  |  |  |
| Fungi, total | 33 (9.1) | 19 (9) | 14 (9.3) | 0.915 |
| Candida albicans | 20 (5.5) | 16 (7.6) | 4 (2.7) | 0.044 |
| Candida spp. other than C. albicans | 11 (3.0) | 2 (0.9) | 9 (6) | 0.015 |
| [aspergillus](javascript:;) | 2 (0.6) | 1 (0.5) | 1 (0.7) | 1.000 |

Note: *P*-value <0.05 indicated statistical significance.

Table S3. Distribution of survivors and non-survivors between APACHE II and SOFA scores

| **Groups** | **APACHE Ⅱ** | | | **SOFA** | | |
| --- | --- | --- | --- | --- | --- | --- |
|  | <16 | 17-24 | >24 | 2-5 | 6-10 | >10 |
| Non-survivors (n, %) | 6 (8.3) | 23 (31.9) | 43(59.7) | 13 (18.1) | 24(33.3) | 35(48.6) |
| Survivors (n, %) | 32(22.2)**^*^** | 89(61.8)**^*^** | 23(16.0)**^*^** | 39(27.1)^∆^ | 80(55.6)^∆^ | 25(17.4)^∆^ |

Data were presented as number (%). ^*, ∆^*P*-value <0.05 compared with the non-survivors.

Table S4. The immune cell counts in patients received glucocorticoids between survivors and non-survivors.

| Parameters | Total | Survivors | Non-survivors | *P* value |
| --- | --- | --- | --- | --- |
|  | (n=51) | (n=10) | (n=41) |  |
| Neutrophil (×10^9^/L) | 10.46±1.79 | 10.66±1.48 | 10.41±1.87 | 0.701 |
| [Lymphocyte](javascript:;) (×10^9^/L) | 1.66±0.59 | 2.43±0.60 | 1.47±0.41 | <0.01 |
| Monocyte (×10^9^/L) | 3.30±0.80 | 3.92±1.09 | 3.15±0.64 | 0.005 |

Note: *P*-value <0.05 indicated statistical significance.
