# Supplementary material for: Predictive value of immune cell counts and neutrophil-to-lymphocyte ratio for 28-day mortality in patients with sepsis caused by intra-abdominal infection
Source: Burns Trauma. 2021 Mar 22;9:tkaa040. doi: 10.1093/burnst/tkaa040 (PMC7982795; doi:10.1093/burnst/tkaa040)
Supplement: Editing_Certificate_tkaa040 [file editing_certificate_tkaa040.pdf]

SPRINGER NATURE

Author Services

Editing Certificate

This document certifies that the manuscript

Predictive value of immune cell counts and neutrophil to lymphocyte ratio for the 28-day mortality in patients with sepsis caused by intra-abdominal infection

prepared by the authors

Shuangqing Liu, Yuxuan Li, Fei She, Xiaodong Zhao, Yongming Yao

was edited for proper English language, grammar, punctuation, spelling, and overall style by one or more of the highly qualified native English speaking editors at SNAS.

This certificate was issued on **August 19, 2020** and may be verified on the SNAS website using the verification code **B970-2E03-80F8-9832-6BE9**.

Neither the research content nor the authors' intentions were altered in any way during the editing process. Documents receiving this certification should be English-ready for publication; however, the author has the ability to accept or reject our suggestions and changes. To verify the final SNAS edited version, please visit our verification page at secure.authorservices.springernature.com/certificate/verify. If you have any questions or concerns about this edited document, please contact SNAS at support@as.springernature.com.

SNAS provides a range of editing, translation, and manuscript services for researchers and publishers around the world. For more information about our company, services, and partner discounts, please visit authorservices.springernature.com.
